# Enhanced Expression of *lmb* Gene Encoding Laminin-Binding Protein in *Streptococcus agalactiae* Strains Harboring IS*1548* in *scpB-lmb* Intergenic Region

**DOI:** 10.1371/journal.pone.0010794

**Published:** 2010-05-24

**Authors:** Rim Al Safadi, Souheila Amor, Geneviève Hery-Arnaud, Barbara Spellerberg, Philippe Lanotte, Laurent Mereghetti, François Gannier, Roland Quentin, Agnès Rosenau

**Affiliations:** 1 Equipe d'Accueil 3854 Bactéries et Risque Materno-Fœtal, Institut Fédératif de Recherche 136 Agents Transmissibles et Infectiologie, UFR Médecine, Université François Rabelais de Tours, Tours, France; 2 Institut für Medizinische Mikrobiologie und Hygiene, Universitäsklinikum Ulm, Ulm, Germany; 3 Unité Mixte de Recherche CNRS FRE 3092 Physiologie des Cellules Cardiaques et Vasculaires, UFR Sciences, Université François Rabelais de Tours, Tours, France; 4 Service de Bactériologie et Hygiène Hospitalière, Hôpital Trousseau, CHRU de Tours, Tours, France; National Institute of Allergy and Infectious Diseases, National Institutes of Health, United States of America

## Abstract

Group B streptococcus (GBS) is the main cause of neonatal sepsis and meningitis. Bacterial surface proteins play a major role in GBS binding to and invasion of different host surfaces. The *scpB* and *lmb* genes, coding for fibronectin-binding and laminin-binding surface proteins, are present in almost all human GBS isolates. The *scpB-lmb* intergenic region is a hot spot for integration of two mobile genetic elements (MGEs): the insertion element IS*1548* or the group II intron GBSi1. We studied the structure of *scpB-lmb* intergenic region in 111 GBS isolates belonging to the intraspecies major clonal complexes (CCs). IS*1548* was mostly found (72.2%) in CC19 serotype III strains recovered more specifically (92.3%) from neonatal meningitis. GBSi1 was principally found (70.6%) in CC17 strains, mostly (94.4%) of serotype III, but also (15.7%) in CC19 strains, mostly (87.5%) of serotype II. No MGE was found in most strains of the other CCs (76.0%), notably CC23, CC10 and CC1. Twenty-six strains representing these three genetic configurations were selected to investigate the transcription and expression levels of *scpB* and *lmb* genes. Quantitative RT-PCR showed that *lmb* transcripts were 5.0- to 9.6-fold higher in the group of strains with IS*1548* than in the other two groups of strains (P<0.001). Accordingly, the binding ability to laminin was 3.8- to 6.6-fold higher in these strains (P≤0.001). Moreover, Lmb amount expressed on the cell surface was 2.4- to 2.7-fold greater in these strains (P<0.001). By contrast, *scpB* transcript levels and fibronectin binding ability were similar in the three groups of strains. Deletion of the IS*1548* sequence between *scpB* and *lmb* genes in a CC19 serotype III GBS strain substantially reduced the transcription of *lmb* gene (13.5-fold), the binding ability to laminin (6.2-fold), and the expression of Lmb protein (5.0-fold). These data highlight the importance of MGEs in bacterial virulence and demonstrate the up-regulation of *lmb* gene by IS*1548*; the increased *lmb* gene expression observed in CC19 serotype III strains with IS*1548* may play a role in their ability to cause neonatal meningitis and endocarditis.

## Introduction

*Streptococcus agalactiae* (group B streptococcus [GBS]) is an important human and bovine pathogen. It is the most common agent of invasive infections in neonates, causing pneumonia, septicemia and meningitis, and is an increasingly common pathogen in adults [1], [2]. GBS species has been divided into phylogenetic lineages by a variety of techniques among which multilocus sequence typing (MLST) has become the standard method [3], [4], [5]. The presence in GBS genome of mobile genetic elements (MGEs) suggests that horizontal genetic transfer may play an important role in genome diversification and in the emergence of virulent clones [6], [7], [8]. It has been demonstrated that GBS diseases are mostly caused by a limited set of clonal lineages [3], [9], [10], [11] and that several MGEs are genetic markers for particular genetic lineages. Indeed, clonal complex (CC) 17 appears to be strongly able to invade the central nervous system (CNS) of neonates and is marked by the group II intron GBSi1, whereas CC19 causes infections among both neonates and adults and is marked by the insertion sequence IS*1548* [12], [13]. This might reflect differences in a number of fitness or virulence factors including the expression of adhesins that have not been studied yet.

**Figure 1:**
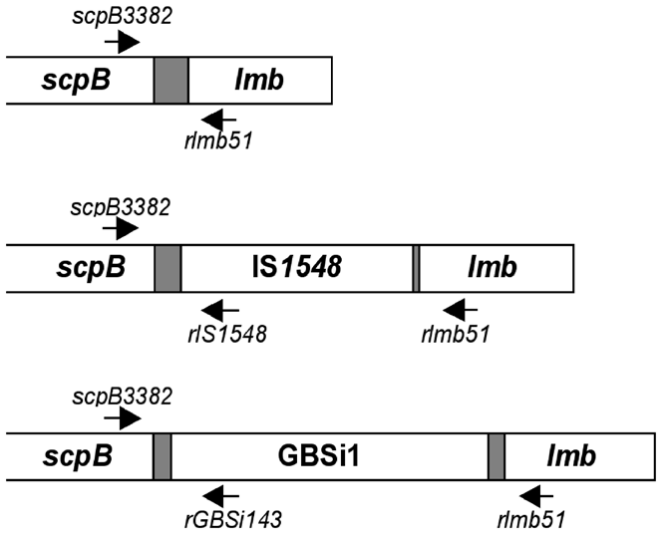
Genomic organization of the *scpB-lmb* intergenic region. Dark grey box represents the noncoding spacer region. Primer set *scpB3382/rlmb51* was used to determine the structure of the *scpB-lmb* intergenic region. The presence of the insertion sequence IS*1548* and of the group II intron GBSi1 in the spacer was then confirmed using primer sets *scpB3382/r1S1548*, and *scpB3382/rGBSi143*.

Genes encoding the surface proteins ScpB and Lmb are located on a composite transposon [14]. Three different structures of the *scpB-lmb* intergenic region are described (Fig. 1) since this region appears to be a hot spot for integration that may contain the two types of MGEs above-mentioned, GBSi1 or IS*1548* [13], [15], [16], [17], [18]. GBSi1 and IS*1548* are located 97-bp upstream and 9-bp upstream of the putative promoter of the *lmb* gene, respectively [17]. In addition to cleavage of the chemotactic complement component C5a, ScpB mediates GBS binding to human immobilized fibronectin, a large dimeric glycoprotein present in the extracellular matrix in a fibrillar form [19]; ScpB also plays a role in the invasion of GBS into epithelial cells [20], and an induced transcription of *scpB* by components of human serum has recently been reported [21]. Binding of GBS to human laminin, a major glycoprotein of the basement membrane [22], is mediated by the surface-associated lipoprotein Lmb (laminin-binding protein) [23]. Lmb shows homology to members of the LraI family that includes Lsp/Lbp of *Streptococcus pyogenes* [23], [24], [25] and it promotes GBS invasion into human brain microvascular endothelial cells [26]. It has not been explored yet if the presence of MGEs in *scpB-lmb* intergenic region correlates with particular levels of transcription and expression of *lmb* and *scpB* genes.

In this study, we analyzed the genetic diversity of the *scpB-lmb* intergenic region in a collection of 111 GBS isolates belonging to the major phylogenetic lineages. Strains representing the three genetic configurations found were selected to determine the level of transcription of *lmb* and *scpB* genes, the ability of strains to bind to human laminin and fibronectin, and the Lmb expression on the bacterial cell surface. We found that i) the structural diversity of the *scpB-lmb* intergenic region was related to particular GBS lineages ii) MGE presence had no influence on *scpB* gene expression and iii) *lmb* gene transcription and expression were significantly higher in strains possessing IS*1548* between *scpB* and *lmb* genes. We then constructed an IS*1548* deleted mutant, and by comparing its properties to those of the wild type parent strain, we demonstrated the role of IS*1548* on the up-regulation of *lmb* gene.

## Results

### Distribution of GBSi1 and IS*1548* in GBS isolates

PCR was performed to characterize the region between *scpB* and *lmb* genes in the 111 GBS clinical isolates, using a forward primer at the 3′end of *scpB* gene (*scpB3382*) and a reverse primer at the 5′end of *lmb* gene (*rlmb51*) (Fig. 1). A 400-bp product indicated the lack of any insert in the spacer region. A 2,200-bp and a 1,600-bp product were obtained in the presence of GBSi1 and of IS*1548*, respectively. The presence of GBSi1 and IS*1548* were then confirmed using primer sets *scpB3382/rGBSi143* and *scpB3382/rIS1548*, respectively (Fig. 1). GBSi1 was found in 51 (45.9%) strains, IS*1548* was found in 18 (16.2%) strains, and none of the two MGEs was observed in 40 (36.0%) isolates and in NEM316 strain. For two strains (V4 and G8) and for *ΔscpB-lmb* NEM316 mutant [14], no amplicons were obtained, and the absence of amplification with primer sets *scpB99/rscpB238* and *lmb76*/*rlmb193* indicated that these strains lacked *scpB* and *lmb* genes.

### Relationship between MLST genetic lineage, clinical origin, distribution of MGEs, and capsular serotype

The 111 isolates were resolved into 40 sequence types (STs), and clonal complexes (CCs) including isolates sharing six or seven identical alleles were defined. The major CCs were CC17 (34.2%), CC19 (18.9%), CC10 (13.6%), and CC23 (10.8%). A relationship between clonal complexes and isolates clinical origin was observed: 28/38 (73.7%) of the CC17 strains and 16/21 (76.2%) of the CC19 strains were recovered from invasive infections of neonates, whereas 7/12 (58.3%) of the CC23 strains and 36/40 (90.0%) of the strains of other CCs were recovered from gastric fluid of colonized asymptomatic neonates and from vaginal swabs of pregnant asymptomatic women. The structure of *scpB-lmb* intergenic region was related to the distribution of strains in clonal complexes (Table 1): 36/38 (94.7%) of the CC17 strains had GBSi1 and 11/12 (91.7%) of the CC23 strains had no MGE; in CC19, 13/21 strains (61.9%) had IS*1548* and the other 8 strains (38.1%) had GBSi1. Of the 13 CC19 strains carrying IS*1548*, 12 (92.3%) originated from invasive infections (Table 2). Finally, 70.6% of the strains that had GBSi1 belonged to CC17 and 72.2% of the strains that had IS*1548* belonged to CC19 (Table 1); all these CC17 strains except 2 and all these CC19 strains were of capsular serotype III (Table 2). On the contrary, seven of the eight strains (87.5%) from CC19 that had GBSi1 were of capsular serotype II (Table 2). The distribution of the 40 strains that had no MGE in the *scpB-lmb* intergenic region was various with 27.5% from CC23, 27.5% from CC10, 15.0% from CC7, 12.5% from CC1, only 5.0% from CC17 and none of them from CC19. A great diversity of capsular serotypes was observed among the strains lacking MGEs with notably 32.5% serotype Ia, mostly from CC23, 25% serotype Ib, mostly from CC10, 12.5% serotype III, and 12.5% serotype II strains.

**Table 1:** Distribution of 109 GBS strains according to the structure of *scpB-lmb* intergenic region and according to clonal complexes as determined by MLST.

		Clonal complexes (CC)						
	N° of strains	CC17	CC19	CC23	CC10	CC7	CC1	Other CC
		N° (%)	N° (%)	N° (%)	N° (%)	N° (%)	N° (%)	N° (%)
	n = 109	n = 38	n = 21	n = 12	n = 15	n = 6	n = 7	n = 10
Without MGE	40	2 (5.0)	0 (0.0)	11 (27.5)	11 (27.5)	6 (15.0)	5 (12.5)	5 (12.5)
GBSi1	51	36 (70.6)	8 (15.7)	1 (2.0)	2 (3.9)	0 (0.0)	2 (3.9)	2 (3.9)
IS*1548*	18	0 (0.0)	13 (72.2)	0 (0.0)	2 (11.1)	0 (0.0)	0 (0.0)	3 (16.7)

**Table 2:** Mobile genetic elements (MGEs) in *scpB-lmb* intergenic region of CC17, CC19, CC23, and CC10 GBS strains from infected and colonized. patients.

CC	Serotype	N° of isolates	MGE	N° of invasive isolates	N° of colonizing isolates
17	Ia	1	GBSi1	1	0
	III	34	GBSi1	27	7
	III	1	no MGE	0	1
	NT^a^	1	GBSi1	0	1
	NT	1	no MGE	0	1
19	II	7	GBSi1	4	3
	III	13	IS*1548*	12	1
	NT	1	GBSi1	0	1
23	Ia	10	no MGE	4	6
	Ia	1	GBSi1	0	1
	III	1	no MGE	1	0
10	Ib	8	no MGE	1	7
	Ib	1	GBSi1	0	1
	II	3	no MGE	0	3
	II	1	GBSi1	0	1
	II	2	IS*1548*	0	2

a NT: not typeable.

### Quantification of *scpB* and *lmb* transcripts

**Figure 2:**
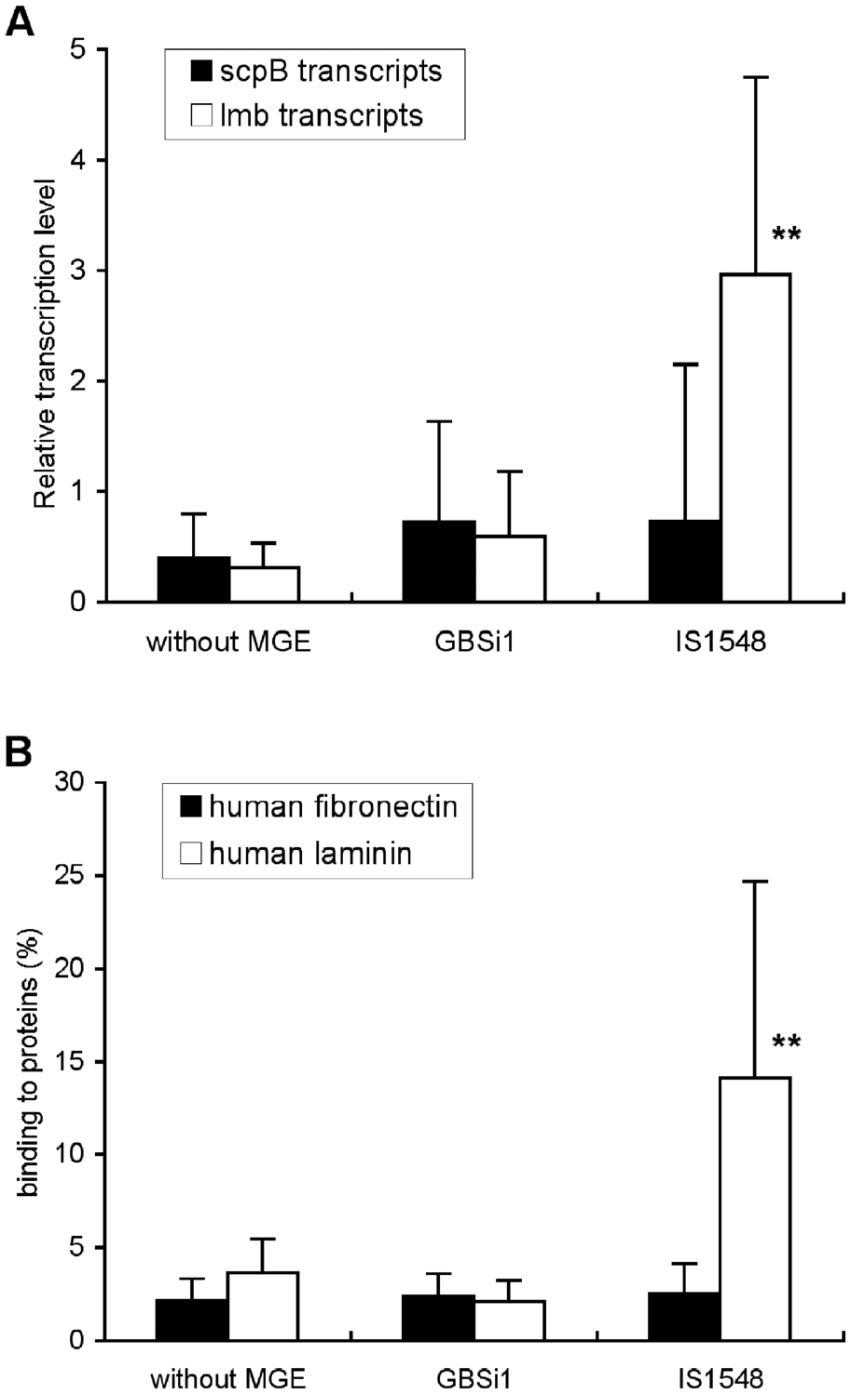
Properties of 26 GBS isolates in relation to the *scpB-lmb* intergenic region structure (8 strains without MGE, 9 strains with GBSi1 and 9 strains with IS*1548*). (A) Relative transcription levels of *scpB* (filled boxes) and *lmb* (open boxes) genes. The amount of transcripts of each gene was normalized to the amount of *gyrA* transcripts and expressed relative to the level of transcription in L19 GBS isolate that has no MGE. Boxes are means and bars are standard deviation of the means of the relative level of transcription in each group of strains. ** The mean transcription level of *lmb* gene was significantly higher in the group of strains with IS*1548* than in the other two groups of strains (*P*<0.001). (B) Binding ability of GBS isolates to immobilized human fibronectin (filled boxes) and to immobilized human laminin (open boxes). Boxes are means and bars are standard deviation of the means of the fibronectin and laminin binding percentages in each group of strains. ** The mean binding ability to laminin was significantly higher in the group of strains with IS*1548* than in the other two groups of strains (*P*≤0.001).

In order to investigate if the presence of GBSi1 and IS*1548* in *scpB-lmb* intergenic region affected the transcription of *scpB* and *lmb* genes, 26 strains representing the three genetic configurations of the various phylogenetic lineages were selected among the 111 isolates: nine strains had GBSi1 and were from CC17 (3 strains), CC19 (5 strains) and CC1 (1 strain); nine strains had IS*1548* and were from CC19 (4 strains), CC10 (2 strains) and three singleton STs (3 strains); eight strains had no MGE and were from CC23 (3 strains), CC10 (3 strains) and CC1 (2 strains). Transcription levels were determined by real time PCR as detailed in Material and Methods. In NEM316 deleted mutant (*ΔscpB-lmb*) and in V4 strain that both lack the *scpB* and *lmb* genes, no transcripts were detected. Figure 2A shows the mean levels of specific gene transcripts in relation to the structure of the *scpB-lmb* intergenic region. The three groups of strains revealed similar transcript levels of *scpB* gene (*P* = 0.96), indicating that the *scpB* gene transcription was not dependant on the intergenic region pattern. On the contrary, the mean transcription level of *lmb* gene in the group of strains with IS*1548* was 5.0- to 9.6-fold higher than that of the groups of strains with GBSi1 and without MGE in *scpB-lmb* intergenic region, respectively, (*P*<0.001 as determined with Kruskal-Wallis test and Dunn's Method for pairwise multiple comparison). The transcription level of *lmb* gene was not related to the phylogeny of strains but to the structure of *scpB-lmb* intergenic region. Indeed, as shown in Table 3, for the nine CC19 strains studied, the mean transcription level of *lmb* gene was 0.86±0.69 for the five strains having GBSi1, and 2.69±1.34 for the four strains having IS*1548*. Similarly, for the five CC10 strains studied, the mean transcription level of *lmb* gene was 0.19±0.16 for the three strains without MGE, and 2.17±0.34 for the two strains having IS*1548*. In the groups of strains with GBSi1 and without MGE, the transcription levels of *scpB* and *lmb* genes were similar (Fig. 2A). By contrast, in strains harbouring IS*1548*, the transcription level of *lmb* gene was significantly higher than that of *scpB* gene (*P* = 0.001).

**Table 3:** *lmb* gene expression in 14 strains from two phylogenetic lineages in relation to the presence of the IS*1548* sequence upstream *lmb* gene.

	CC19		CC10	
	IS*1548* +	IS*1548* −	IS*1548* +	IS*1548* −
	n = 4	n = 5	n = 2	n = 3
*lmb* gene transcripts^a^	2.69±1.34	0.86±0.69	2.17±0.34	0.19±0.16
Laminin binding (%)^b^	9.36±2.25	2.60±1.06	12.84±6.74	2.52±1.10
Lmb protein expression^c^	6.01±2.70	2.50±0.70	4.80±1.50	1.45±0.20

a *lmb* gene transcripts were quantified by real time RT-PCR. The amount of *lmb* transcripts was normalized to the amount of *gyrA* transcripts and expressed relative to the level of transcription in L19 GBS isolate that has no MGE.

b The percentage of binding to immobilized human laminin was obtained by the ratio between the number of bound bacteria and the number of bacteria present in the inoculum.

c Lmb protein expressed at the bacterial cell surface was quantified in an ELISA-type assay, as described in Figure 3.

### Binding of GBS strains to immobilized human fibronectin and laminin

In order to analyze if the expression of *scpB* and *lmb* genes was correlated with the level of transcription, we tested the ability of the 26 GBS strains to bind to human fibronectin and to human laminin (Fig. 2B). No difference between the fibronectin binding ability of the three groups of strains was observed (*P* = 0.86). By contrast, strains with IS*1548* bound significantly more strongly to laminin (14.13%±10.55%) than strains that had GBSi1 (2.14%±1.13%) and strains without MGE in the *scpB-lmb* intergenic region (3.70%±1.78%) (*P*≤0.001 as determined with Kruskal-Wallis test and Dunn's Method for pairwise multiple comparison). The mean binding ability to human laminin of strains with IS*1548* was thus 3.8- to 6.6-fold increased as compared with strains without MGE and with GBSi1, respectively. As observed for the transcription level of *lmb* gene, the binding ability to laminin was not related to the phylogenetic lineages of strains, but to the structure of *scpB-lmb* intergenic region. Indeed, as shown in Table 3, for the nine CC19 strains studied, the mean binding ability to laminin was 2.60%±1.06% for the five strains having GBSi1, and 9.36%±2.25% for the four strains having IS*1548*. Similarly, for the five CC10 strains studied, the mean binding ability to laminin was 2.52%±1.10% for the three strains without MGE, and 12.84±6.74% for the two strains having IS*1548*.

### Quantification of Lmb on bacterial cell surface

To further analyze if the stronger binding ability to laminin of strains with IS*1548* was in relation with a stronger expression of the laminin binding protein Lmb, quantification of Lmb on the bacterial surfaces of the 26 GBS strains was performed in an ELISA-type assay using polyclonal rabbit antibodies raised against Lmb. Preimmune rabbit serum did not react with GBS strains. As depicted in Fig. 3, the amount of Lmb was 2.4- to 2.7-fold increased in GBS isolates with IS*1548* as compared with the GBS strains with GBSi1 or without MGE in *scpB-lmb* region, respectively (*P*<0.001 as determined by ANOVA with Fisher's post-hoc test). These findings support our results of *lmb* transcripts quantification and of binding ability of strains to laminin and point out the association of IS*1548* with the induction of *lmb* gene expression.

**Figure 3:**
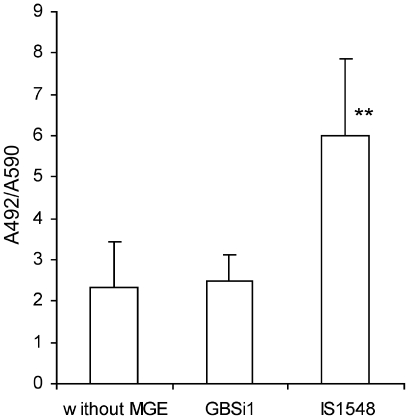
Expression of Lmb protein on GBS cells in relation to the *scpB-lmb* intergenic region structure. Lmb protein was quantified in an ELISA-type assay. GBS cells (8 strains without MGE, 9 strains with GBSi1, 9 strains with IS*1548*), were immobilized in microtiter wells and incubated with rabbit anti-Lmb antibodies. Binding of antibodies to the Lmb protein was quantified by addition of peroxidase-conjugated goat anti-rabbit IgG, and subsequent addition of OPD peroxidase substrate. Boxes are means and bars are standard deviation of the means of the absorbance at 492 nm (A_492_) per adhering bacteria in each group of strains. ** The amount of Lmb protein was significantly higher in the group of strains with IS*1548* as compared with the other two groups of strains (*P*<0.001).

### Properties of the isogenic IS*1548* deleted strain

**Figure 4:**
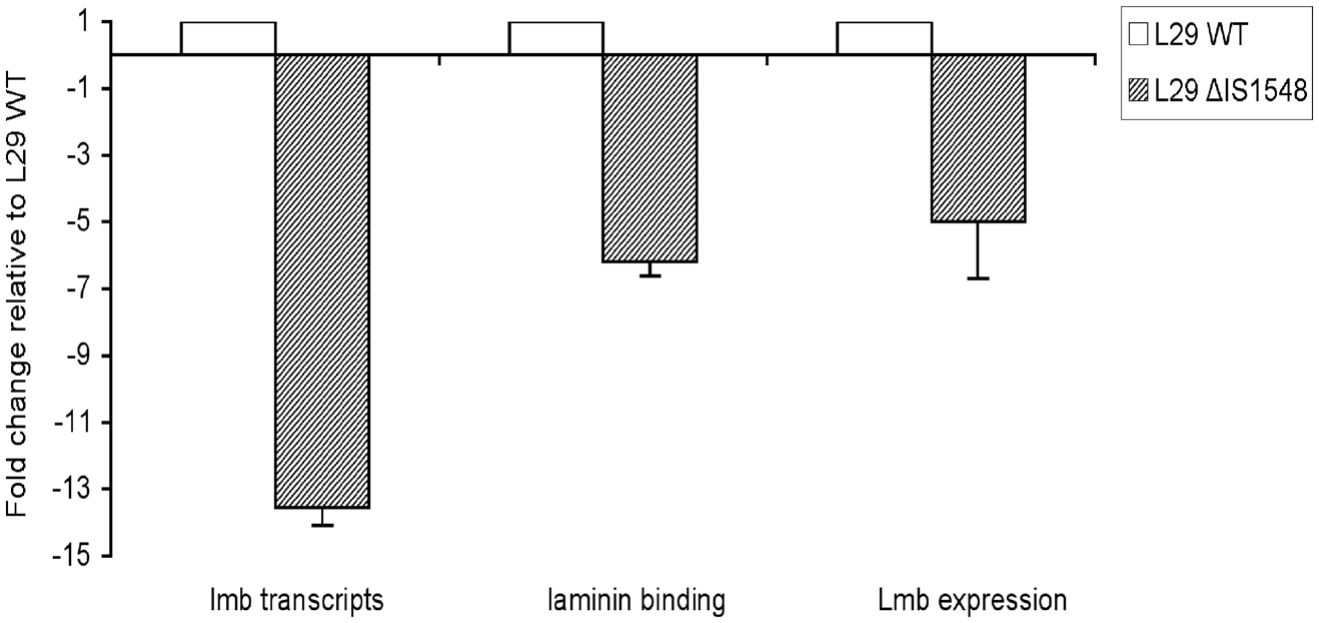
Properties of L29 wild type GBS strain and isogenic IS*1548* deletion mutant. Relative transcription levels of *lmb* gene, binding ability of GBS cells to immobilized human laminin, and expression of Lmb protein on GBS cells as determined by ELISA, in an isogenic mutant deleted for IS*1548* sequence between *scpB* and *lmb* genes (L29*Δ*IS*1548*) (striped boxes) as compared to the parent strain (L29 WT) (open boxes). Each experiment was performed at least three times. Boxes are means and bars are standard deviation of the means.

**Figure 5:**
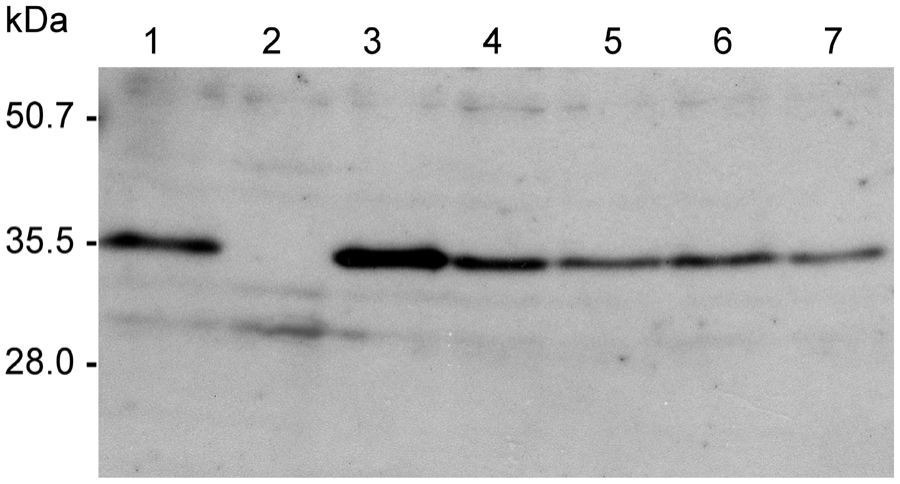
Detection of Lmb protein by Western blot in L29 wild type GBS strain and isogenic IS*1548* deletion mutants. 10 µg of total bacterial cell proteins isolated from strains 1: NEM316 (no MGE), 2: Δ*scpB-lmb* NEM316 mutant, 3: L29 wild type (IS*1548*), 4 to 7: four independent L29*Δ*IS*1548* mutant strains were separated by SDS PAGE and transferred to an Immobilon P polyvinylidene difluoride membrane. The blots were probed with rabbit anti-Lmb antibodies that were detected with a horseradish peroxidase labeled secondary anti-rabbit antibody that was then visualized by the ECL system (Amersham Biosciences). Molecular sizes in kDa are depicted at the left.

In order to determine whether the up-regulation of the *lmb* gene is caused by the upstream copy of IS*1548*, we deleted the IS*1548* sequence between *scpB* and *lmb* genes in the genome of ST-19 serotype III L29 strain. The successful deletion was confirmed by DNA sequencing of a 1.2 kb-PCR product obtained with *scpB2987/rlmb497* primer set (data not shown). L29 wild type (WT) strain and the isogenic mutant (*Δ*IS*1548*) were subsequently tested for transcription of *lmb* gene by quantitative RT-PCR, expression of Lmb protein on cell surface by ELISA and Western blot, and binding ability to laminin. As depicted in Fig. 4, the isogenic mutant showed a 13.5-fold decreased level of *lmb* transcripts, a 6.2-fold decreased ability to bind to laminin, and a 5.0-fold decreased Lmb protein amount as determined by ELISA, as compared to the WT strain. Similarly, detection of Lmb protein by Western blotting (Fig. 5) showed a decreased expression in L29*Δ*IS*1548* as compared to the WT strain.

## Discussion

Mobile genetic elements are an important source of genetic plasticity in prokaryotes. They drive bacterial evolution and adaptation via recombination and horizontal transfer and they influence the virulence of bacteria by modulating gene expression [27]. ScpB and Lmb are two ubiquitous proteins localized at the surface of GBS cells, and are thus good candidates for a GBS vaccine. Therefore, it is of importance to better explore their expression level, especially on account of the genetic diversity of *S. agalactiae* species that is composed of multiple major phylogenetic lineages. In line with this, we screened a large collection of strains representing the diversity of the species for the presence of two MGEs, GBSi1 and IS*1548*, between *scpB* and *lmb* genes, and studied the expression of *scpB* and *lmb* genes for each *scpB-lmb* intergenic region pattern. Moreover, we examined the effect of deleting the upstream IS*1548* copy on *lmb* gene expression in one GBS isolate.

In our collection of 111 strains, only two strains lacked the *scpB* and *lmb* genes, a result in accordance with previous data that had revealed that almost all human isolates contained the *scpB* and *lmb* genes, whereas these genes were absent in a majority of strains of bovine origin [14], [15], [16], [28]. Therefore, our results reinforce the hypothesis that *scpB-lmb* region may be essential for the colonization or infection in humans [14], [21]. As previously reported, we found a mutual exclusiveness of IS*1548* and GBSi1 [9], [12], [17], and a diversity of patterns of the intergenic region in relation to several major GBS lineages: CC17 was characterized by GBSi1 and CC19 was characterized by IS*1548*, while CC23 lacked GBSi1 and IS*1548* [8], [12], [13], [29]. Nevertheless, the CC19 strains clustered into two groups: all the serotype III strains had IS*1548* and all the serotype II strains had GBSi1. Therefore, as previously suggested [9], [30], these two clusters seem to form distinct and relatively homogeneous lineages that could have notable differences in virulence factors expression.

Our different experimental approaches demonstrated that the presence of GBSi1 or IS*1548*, which are located downstream of the *scpB* gene, did not modify *scpB* transcription and expression. By contrast, IS*1548* was associated with an enhanced expression of the *lmb* gene which resulted in a 5.0- to 9.6-fold increased *lmb* transcription, a 3.8- to 6.6-fold increased binding ability to human laminin, and a 2.4- to 2.7-fold increased expression of Lmb protein on the bacterial cell surface, as compared to strains without MGE or with GBSi1. These variations were independent of the phylogenetic position of strains indicating that the *lmb* gene expression was probably directly affected by the IS element acquisition rather than by other genetic events linked to the emergence of particular intraspecies clones. This hypothesis was then demonstrated by construction of an IS*1548* deleted mutant which showed a 13.5-fold decreased transcription of *lmb* gene, a 5.0-fold decreased expression of Lmb protein, and a 6.2-fold reduced binding ability to laminin, as compared to the parent strain. Gene activation following integration of IS elements is a well-known phenomenon, notably described for antibiotic resistance, bacterial metabolism and virulence [31], [32], [33], [34], [35]. Several mechanisms could explain the role of IS*1548* in the up-regulation of the *lmb* gene. First, it could result from the provision of an efficient promoter, carried either entirely by the IS element, or generated as a hybrid structure between IS and the target sequence. Indeed, in *Lactococcus lactis*, a hybrid promoter composed of a −35 sequence located at the right end of an IS-like element, and a −10 sequence located in the region adjacent to the element, created a new promoter which was responsible for the activation of the *citQRP* operon involved in the transport of citrate [32]. Additionally, in *Salmonella enterica* serovar Typhimurium, new promoter sequences were created by the integration of the IS*1* element upstream of the original promoter for the multidrug efflux *acrEF* operon, therefore providing an alternative promoter which activated its expression [33]. The up-regulation of the *lmb* gene could also result from the disruption or displacement of a repressor binding site following the insertion of IS*1548* upstream of *lmb* gene, thus leading to the increase of *lmb* expression. This mechanism was suggested to explain the activation of the endogenous *Bacteroides fragilis* cephalosporinase gene, *cepA*, by IS*1224* [34]. Additional studies are needed to explore these mechanisms.

Genes encoding GBS surface and secreted proteins constitute potentially useful virulence factors to explain the ability of GBS to invade the central nervous system (CNS) of neonates. Several studies have reported that a particular allelic form of *gbs2018*, *srr-2*, and *fbsB* genes encoding peptidoglycan anchored and fibrinogen-binding proteins were specific of the highly virulent ST-17 clone [28], [36], [37], mostly prevalent in neonatal meningitis [5], [13], but no specific factors appeared to be related to the other virulent clonal complexes, especially to CC19 that was regularly described in neonatal meningitis. We have recently reported that 100% of the CC17 strains of the collection used in this study belonged to the group of strains that possessed both *fbsA* and *fbsB* fibrinogen-binding genes and had the highest ability to bind to human fibrinogen [38]; nearly all these strains had GBSi1 in the *scpB-lmb* intergenic region, a configuration that does not increase *lmb* expression. By contrast, we have previously found that 84.0% of the CC19 strains studied here were devoid of the *fbsB* gene *sensu stricto* and had a truncated form of the *fbsA* gene leading to a low fibrinogen binding ability [38]. Therefore, contrarily to CC17 strains, the fibrinogen binding ability does not appear as a major factor to explain the ability of CC19 strains to invade neonatal CNS. Interestingly, 13 of the 21 CC19 strains (61.9%) had IS*1548* in the *scpB-lmb* intergenic region and had consequently an increased binding ability to laminin, and 12 of these 13 CC19 IS*1548* positive isolates were recovered from CNS. It was previously demonstrated that FbsA and Lmb proteins are important factors for GBS adherence to and invasion into the blood-brain barrier that facilitates the entry of the bacteria into the cerebrospinal fluid [26], [39]. Therefore, we may hypothesize that the two fibrinogen binding genes *fbsA* and *fbsB* promote the ability of CC17 strains to invade CNS of neonates whereas the enhanced expression of *lmb* gene due to I*S1548* promotes that of CC19 isolates. Similarly, IS*1548* was identified in the hyaluronidase gene *hylB* of *S. agalactiae* endocarditis isolates [40]. In such strains, an additional copy of IS*1548* was always found downstream of the *scpB* gene [40], [41], [42]. As streptococci colonize cardiac valves with pre-existing endothelial lesions that lead to the exposure of underlying basement membrane structures [43], the enhancement of GBS adhesion to basement membrane laminin may be critical for the bacterial colonization of damaged epithelium and endothelium.

In summary, our results support the importance of transposable elements in bacterial virulence. We demonstrated that IS*1548* strongly activates *lmb* gene expression. This might explain the particular ability of some *S. agalactiae* strains, mostly belonging to CC19 and to capsular serotype III, to invade the CNS of neonates. Further studies are needed to clarify the mechanisms explaining the overexpression of *lmb* gene by IS*1548* in these particular strains.

## Materials and Methods

### Bacterial strains and growth conditions

A total of 111 unrelated human strains representing the genetic diversity of *Streptococcus agalactiae* species were included in the present study: 54 strains from the cerebrospinal fluid of infected neonates, 21 strains from the gastric fluid of colonized asymptomatic neonates and 36 strains from vaginal swabs of colonized asymptomatic pregnant women. Strains were isolated between 1986 and 1990 from 25 general hospitals throughout France [11]. Neonatal specimens were obtained from infected neonates suffering of meningitis and from non infected neonates with risk factor, and vaginal specimens were obtained from asymptomatic pregnant women during the process of routine clinical diagnostic procedures, as part of the usual prenatal and postnatal screening. All data were analyzed anonymously. According to the information we obtained from the Institutional Review Board (more than 20 years ago), this type of study did not require an ethics approval and the patient consent. All strains had previously been serotyped on the basis of capsular polysaccharides [11]. Six serotypes were identified i.e. serotypes Ia (16 strains), Ib (12 strains), II (17 strains), III (55 strains), IV (3 strains), and V (3 strains), and five strains were not typeable. *S. agalactiae* wild-type NEM316, and an isogenic *scpB-lmb* mutant of NEM316, which comprises a deletion of the complete *scpB-lmb* composite transposon structure (*ΔscpB-lmb*) were used as control strains. The strain was generated by insertion of a pG^+^host5 plasmid [44] into the *lmb* gene of NEM316, and subsequent mobilization of the plasmid as described by Franken et al. [14].

GBS strains were stored at −80°C in Schaedler-vitamin K_3_ broth (bioMérieux, Marcy l'Etoile, France) with 10% glycerol. The bacteria were grown for 24 h on 5% horse blood Trypticase soja (TS) agar plates (bioMérieux) at 37°C.

*Escherichia coli* DH5α was used for cloning purposes. It was grown at 37°C in Luria Broth. *E. coli* and GBS clones carrying the pG^+^host5 plasmid were selected in the presence of 300 µg/ml and 2 µg/ml erythromycin, respectively.

### Construction of an IS1548 deletion mutant

The IS*1548* sequence was deleted in the chromosome of ST-19 serotype III L29 GBS strain by a method adapted from that described by Schubert et al. [45]. Briefly, a DNA fragment including 421 bp at the 3′ end of *scpB* gene, the whole *scpB-lmb* intergenic region, and 400 bp at the 5′ end of *lmb* gene was amplified by PCR with the primer pair *IS1548*_del1 5′-CCGCGGATCCGGCCACTCTAATAAGCCAGAA and *IS1548*_del2 5′-GGGGGTACCAATGCCTTGTGTGACTTCCA from the genome of ST-23 serotype III V27 GBS strain that harbors no MGE in this region. The *Bam*HI restriction site of *IS1548*_del1 and the *Kpn*I restriction site of *IS1548*_del2 are underlined. The resulting PCR product and the thermosensitive vector pG^+^host5 [44] were digested with *Bam*HI and *Kpn*I, ligated, and transformed into *E. coli* DH5α. The resulting plasmid pG^+^ *IS1548neg* was electroporated into *S. agalactiae* L29 [46], and transformants were grown at 28°C with erythromycin selection. Cells in which pG^+^ *IS1548neg* had integrated into the chromosome were selected at 37°C under erythromycin pressure. Four of such clones were serially passaged for 6 days in Todd-Hewitt (TH) broth (Sigma, St Quentin Fallavier, France) at 28°C without erythromycin pressure to facilitate the excision of plasmid pG^+^ *IS1548neg*, leaving the desired IS*1548* deletion in the chromosome. Dilutions of the serially passaged cultures were plated onto TS agar plates, and single colonies were tested for erythromycin sensitivity to identify pG^+^ *IS1548neg* excisants. Successful deletion of IS*1548* sequence was confirmed by PCR using *scpB2987/rlmb497* primer set (Table 4) and by sequencing the amplified fragment.

**Table 4:** Oligonucleotide primers.

Name of primer	Sequence (5′-3′)	Target sequence
*scpB3382*	TTATGACCACTTTCTTCTTGGGA	*scpB-lmb* intergenic region
*rlmb51*	CTATCATTACTAAACTCACAAC	
*scpB2987*	AATGGAAGGCGCTACTGTTC	*scpB-lmb* intergenic region
*rlmb497*	GGATCCAAACGTCCTAGCTC	
*scpB3382*	TTATGACCACTTTCTTCTTGGGA	IS*1548* in *scpB-lmb* intergenic region
*rIS1548*	CTTCATCCTTTTGTGCCCGGACATC	
*scpB3382*	TTATGACCACTTTCTTCTTGGGA	GBSi1 in *scpB-lmb* intergenic region
*rGBSi143*	CTCCATTTCCTCATCGTCAG	
*lmb76*	CCCAAACAGCCTACGCAAG	*lmb* gene
*rlmb193*	TGCCTGCACCTGATTGGATC	
*scpB99*	TGTGACAGAAGACACTCCTG	*scpB* gene
*rscpB238*	CGTCATCTGCTACTGTTTCTCC	
*gyrA*	CGGGACACGTACAGGCTACT	*gyrA* gene
*rgyrA*	CGATACGAGAAGCTCCCACA	

### PCR

All primers used (Table 4) were purchased from Eurogentec (Seraing, Belgium). Bacterial genomic DNA extracted and purified by conventional methods [47] was used as the template for PCR assays. PCR was carried out with 0.5 U *Taq* DNA polymerase (Roche Diagnostics, Mannheim, Germany) with an initial 5 min hold at 94°C followed by 30 cycles of amplification steps of 1 min at 94°C, 0.5 min at 55°C, and 2 min at 72°C, followed by a final 10 min step at 72°C.

### Quantification of specific transcripts with real-time PCR

*S. agalactiae* strains were grown overnight in 50 ml of TH broth to stationary growth phase ([OD_595_] = 1.2). Bacterial cells pelleted by centrifugation were lysed mechanically with 0.25–0.5 mm glass beads (Sigma) in Tissue Lyser (Qiagen, Hilden, Germany) for 6 min at 30 Hz. RNA was purified by using the RNeasy Mini kit (Qiagen), then treated with DNase using DNAfree kit (Ambion, Cambridgeshire, UK) and checked for DNA contamination by PCR amplification without prior reverse transcription. As no amplicons were obtained, the possibility of DNA contamination during RNA preparation could be excluded. Reverse transcription of 1 µg of RNA was performed with random hexanucleotides and the Quantiscript Reverse Transcription kit (Qiagen). Real-time quantitative PCR was performed in a 25 µl reaction volume containing cDNA (50 ng), 12.5 µl QuantiTect SYBR Green PCR Master Mix (Qiagen), and 0.3 µM of each gene-specific primer in an iCycler iQ detection system (BioRad). The specific primers for *gyrA*, *lmb* and *scpB* genes listed in Table 4 were used. The PCR consisted of an initial 15 min hold at 95°C followed by 40 cycles, each of 15 sec at 94°C, 30 sec at 58°C, and 30 sec with fluorescence acquisition at 72°C. The specificity of the amplified product was verified by generating a melting-curve with a final step of 50 cycles of 10 sec at an initial temperature of 70°C, increasing 0.5°C each cycle up to 95°C. The quantity of cDNA for the investigated genes was normalized to the quantity of *gyrA* cDNA in each sample. The *gyrA* gene was chosen as an internal standard since gyrase genes represent ubiquitously expressed house-keeping genes that are frequently used for the normalization of gene expression in quantitative reverse transcription-PCR experiments [48], [49]. The transcription levels of *scpB* and *lmb* genes in the GBS isolate L19 which has no MGE in the *scpB-lmb* intergenic region, were treated as the basal levels. Each experiment was performed at least three times.

### Binding of GBS to immobilized human fibronectin and human laminin

All binding assays were performed in triplicate. Flat bottomed 96-well polystyrene plates (Ciblex, Ivry sur Seine, France) were coated for 18 h at room temperature with 10 µg/ml human plasma fibronectin (Sigma) or with 10 µg/ml human placenta laminin purified by immunoaffinity chromatography (Sigma) diluted in phosphate-buffered saline (PBS) (150 mM NaCl, 10 mM sodium phosphate buffer, pH 7.2). Bacterial cells were harvested from overnight cultures in TH broth and resuspended in PBS. Laminin- and fibronectin-coated wells were washed, and then 50 µl of PBS containing 1×10^5^ to 1×10^6^ CFU per ml were added to each well. After incubation for 90 min at 37°C, non binding bacteria were removed by washing with PBS. Bound bacteria were subsequently unbound by the addition of a 0.01% solution of protease/serine protease mix (Sigma) to each well as previously described [38], then the viable bacteria were quantified by plating serial dilutions onto TS agar plates. The percentage of binding to human laminin and fibronectin was obtained by the ratio between the number of bound bacteria and the number of bacteria present in the inoculum.

### Quantification of Lmb protein on bacterial cells by ELISA

Quantification of Lmb was determined in an ELISA-type assay. Flat bottomed 96-well polystyrene plates were coated overnight at 4°C with GBS cells grown to stationary phase (5×10^5^ CFU/well). After washing with PBS, the wells were treated for 1 h at room temperature with 2% bovine serum albumin in PBS in order to block additional protein binding sites. The wells were washed with 0.05% Tween 20 in PBS and incubated 1 h at 37°C with polyclonal rabbit anti-Lmb antibodies which were raised against Lmb recombinant protein [23], or with rabbit preimmune serum at a dilution of 1∶200. The absence of reactivity of preimmune serum and the specificity of anti-Lmb antibodies towards Lmb protein were previously shown [23], and the ability of anti-Lmb antibodies to inhibit bacterial binding to laminin was demonstrated [26]. After washing, wells were incubated for 1 h at 37°C with a peroxidase-conjugated goat anti-rabbit IgG (Camarillo, CA, USA) at a dilution of 1∶10,000. The conjugated enzyme was allowed to react with *o*-phenylenediamine dihydrochloride (OPD) (Sigma) and absorbance at 492 nm (A_492_) was monitored with a microtiter plate reader (Labsystems iEMS Reader MF, Labsystems, Helsinki, Finland). ELISA results were expressed as A_492_ values divided by the number of bacteria that had adhered to each well, as determined by crystal violet staining, a common method used to quantify bacteria adhering to plastics, more particularly in biofilms [50], [51], [52]: cells were stained with 100 µl 0.3% crystal violet at room temperature for 15 min. Excess crystal violet was then discarded and the wells were washed 3 times with water. Crystal violet was then extracted from the cells by 200 µl absolute ethanol for 45 min at room temperature and transferred to a new microtiter plate. The quantity of bacteria was estimated by measuring the A_590_ of each well using a microtiter plate reader.

### Detection of Lmb protein by Western blot

Total bacterial cell protein was isolated from GBS strains as previously described [53]. For each strain, 10 µg of protein was separated on a 12% SDS polyacrylamide gel and transferred to an Immobilon P polyvinylidene difluoride membrane. Following preabsorption of the antibody with a an Lmb negative *S. agalactiae* strain, the blots were probed with polyclonal rabbit anti-Lmb antibodies [23] at a dilution of 1∶200. For the detection of bound primary antibody, a horseradish peroxidase labeled secondary anti-rabbit antibody was used at a dilution of 1∶8,000. Bound secondary antibody was visualized by the ECL system (Amersham Biosciences Europe, Freiburg, Germany) in accordance with instructions provided by the manufacturer.

### Multilocus sequence typing

The 111 strains were typed by multilocus sequence typing (MLST) by sequencing ∼500-bp fragments of seven housekeeping genes (*adhP*, *pheS*, *atr*, *glnA*, *sdha*, *glcK*, and *tkt*) as described by Jones et al. [5]. Nucleotide sequences were compared with those available on the MLST database (http://pubmlst.org/sagalactiae/). After assigning an allele number to each locus, the sequence type (ST) which takes in account the allele combination of the seven loci was determined for each isolate. Clonal complexes including isolates sharing six or seven identical alleles were defined.

### Statistical analysis

Data were analyzed using one-way analysis of variance (ANOVA) with Fisher's post-hoc test. When conditions of normality were not met to use parametric analysis, a non-parametric ANOVA test (Kruskal–Wallis) with pairwise multiple comparison procedures (Dunn's Method) was used to find statistical differences between results. Statistically significant difference was determined at 95% confidence level. Parametric tests were carried out with Minitab version 10.51 (MINITAB Inc., State College, PA, USA) and non-parametric tests with SigmaStat version 2.03 (Systat Software Inc., Richmond, CA, USA).
